# Hookah smoking is strongly associated with diabetes mellitus, metabolic syndrome and obesity: a population-based study

**DOI:** 10.1186/s13098-018-0335-4

**Published:** 2018-04-19

**Authors:** Sara Saffar Soflaei, Susan Darroudi, Maryam Tayefi, Abolfazl Nosrati Tirkani, Mohsen Moohebati, Mahmoud Ebrahimi, Habibollah Esmaily, Seyed Mohammad Reza Parizadeh, Ali Reza Heidari-Bakavoli, Gordon A. Ferns, Majid Ghayour-Mobarhan

**Affiliations:** 1 Neurogenic Inflammation Research Center, Department of Modern Sciences and Technologies, Mashhad University of Medical Sciences, Mashhad, Iran; 2 Department of Modern Sciences and Technologies, Mashhad University of Medical Sciences, Mashhad, Iran; 3 Biochemistry of Nutrition Research Center, Faculty of Medicine, Mashhad University of Medical Sciences, Mashhad, Iran; 4 Department of Biochemistry, Faculty of Medicine, Mashhad University of Medical Sciences, Mashhad, Iran; 5 Cardiovascular Research Center, Faculty of Medicine, Mashhad University of Medical Sciences, Mashhad, Iran; 6 Department of Biostatistics and Epidemiology, School of Health, Mashhad University of Medical Sciences, Mashhad, Iran; 7 Division of Medical Education, Brighton and Sussex Medical School, University of Brighton, Rm 342, Mayfield House, Brighton, BN1 9PH UK; 8 Metabolic Syndrome Research Center, School of Medicine, Mashhad University of Medical Sciences, 99199-91766 Mashhad, Iran

**Keywords:** Smoking, Cigarette, Hookah, Water-pipe, Metabolic syndrome, Diabetes, Dyslipidemia, Biochemical measurements

## Abstract

**Objectives:**

The adverse effects of cigarette smoking have been widely studied before, whilst the effects of hookah smoking has received less attention, although it is a common habit in the Middle East. Here we have investigated the effects of cigarette and hookah smoking on biochemical characteristics in a representative population sample derived from the Mashhad stroke and heart atherosclerotic disorder (MASHAD) cohort study, from Northeastern Iran.

**Study design:**

A total of 9840 subjects from the MASHAD population study were allocated to five groups; non-smokers (6742), ex-smokers (976), cigarette smokers (864), hookah smokers (1067), concomitant cigarette and hookah smokers (41).

**Methods:**

Baseline characteristics were recorded in a questionnaire. Biochemical characteristics were measured by routine methods. Data were analyzed using SPSS software and p < 0.05 was considered significant.

**Results:**

After adjustment for age and sex; the presence of CVD, obesity, metabolic syndrome, DM and dyslipidemia were significantly (p < 0.001) related to smoking status. After multivariate analysis, HDL (p < 0.001), WBC (p < 0.001), MCV (p < 0.05), PLT (p < 0.01) and RDW (p < 0.001), and the presence of CVD (p < 0.01), obesity (p < 0.001), metabolic syndrome (p < 0.05) and DM (p < 0.01) remained significant between cigarette smokers and non-smokers. Between hookah smokers and non-smokers; uric acid (p < 0.001), PLT (p < 0.05) and RDW (p < 0.05), and the presence of obesity (p < 0.01), metabolic syndrome (p < 0.001), diabetes (p < 0.01) and dyslipidemia (p < 0.01) remained significant after logistic regression.

**Conclusion:**

There was a positive association between hookah smoking and metabolic syndrome, diabetes, obesity and dyslipidemia which was not established in cigarette smoking.

## Background

Tobacco smoking is one of the leading causes of mortality and morbidity globally. Tobacco smoking is responsible for 6 billion deaths per year globally, and nearly 10% of deaths due to tobacco smoking are in passive smokers [1]. On average, a smokers life span is reduced by 10 years compared to non-smokers [2]. There are different ways of smoking substances including bidis (the hand-rolled cigarette), cigar, cigarettes, roll-your-own, hookah, kretek, pipe smoking and vaporizers [1].

The hookah is the second most common way of smoking tobacco. The hookah, which is also called the “waterpipe” or “shisha” is an apparatus invented in 16th century in an attempt to purify tobacco smoke by passing it through water [3]. Hookah use has become prevalent particularly, in developing countries. The misconception that inhaling smoke through the hookah is less toxic, and its relative cheap price may be responsible for this increase [4]. However, a recent study has indicated that hookah smoking is equivalent to smoking of a cigarette [5].

Although the effects of cigarette smoking on health has been well studied, the effects of hookah smoking on disease development, and biochemical measurements is less studied. Findings on the adverse effects of hookah smoking on lipid profile are inconsistent [6, 7]. Only an experimental study and a single study with a relatively small sample size have been conducted about hookah smoking and hematological measurements [8, 9]. The only study on the effect of hookah smoking and serum glucose level in a small sample population was recently published [10].

We aimed to determine the association between cigarette and hookah smoking and metabolic parameters, obesity, cardiovascular disease, diabetes mellitus, metabolic syndrome and dyslipidemia in the MASHAD study population [11].

## Methods

Data was taken from MASHAD (Mashhad stroke and heart atherosclerotic disorder) study (2010–2012). Individuals were recruited from the population living in Mashhad using a stratified cluster random sampling technique [11]. A total of 9840 participants aged between 35 and 65 entered the study. Baseline characteristics were recorded in a questionnaire including demographic data, history of smoking (cigarette, hookah), cardiovascular risk factors and anxiety and depression tests. Fasting blood samples were taken after 14 h of fasting from ante-cubital vein. Cell blood count (CBC) including hemoglobin (Hb), red blood cell (RBC), mean corpuscular volume (MCV), mean corpuscular hemoglobin (MCH), mean corpuscular hemoglobin concentration (MCHC), hematocrit (HCT), red blood cell distribution width (RDW) and platelet count (PLT) were measured with Sysmex K21.

Levels of fasting blood glucose (FBG), serum triglyceride (TG), total cholesterol, high density lipoprotein (HDL), low density lipoprotein (LDL) and uric acid, serum urea, creatinine (Cr) and hs-CRP were measured by auto-analyzer using Pars Azmoon kits (mg/dl). Glomerular filtration rate (GFR) was calculated using the Cockcroft and Gault formula.

### Definitions

CVD was considered to be present if there was a positive personal history of cardiovascular disease.

Diabetes mellitus was defined as a FBG ≥ 126 mg/dl, or being treated with an oral hypoglycemic agents or insulin.

Hypertension was diagnosed in individuals with systolic blood pressure at or above 140 mmHg and/or a diastolic blood pressure at or above 90 mmHg, or in persons who were on anti-hypertension medication.

The presence of metabolic syndrome was determined using the IDF criteria [12].

Dyslipidemia was defined as TC ≥ 200 mg/dl (5.18 mmol/l), LDL-C ≥ 130 mg/dl (3.36 mmol/l), or TG ≥ 150 mg/dl (1.69 mmol/l), or HDL-C < 40 mg/dl (1.03 mmol/l) in men and < 50 mg/dl (1.30 mmol/l) in women [13].

### Statistical analysis

Statistical analysis was carried out using SPSS version 20 on 2015. ANCOVA, independent sample t test, Chi square and multiple logistic regression tests were carried on for analysis. p < 0.05 was considered significant.

## Results

Out of 9840 participants 6742 were non-smoker, 976 were ex-smoker, 864 were cigarette smoker, 1067 were hookah smoker and 41 were both cigarette and hookah smoker. Data were analyzed after adjustment for age and gender. The association between biochemical measurements and smoking status are shown in Table 1. FBG (p = 0.013), cholesterol (p = 0.045), HDL (p < 0.001), GFR (p < 0.001), uric acid (p < 0.001) and hs-CRP (p < 0.001) were significantly different while LDL (p = 0.9), triglycerides (p = 0.125), BUN (p = 0.63) and Cr (p = 0.65) were the same between the 5 groups defined by smoking status.

**Table 1:** Association between smoking status and biochemical measurements (adjusted for age and sex)

Groups	No smoker (n: 6742)	Ex-smoker (n: 976)	Cigarette smoking (n: 864)	Hookah smoking (n: 1067)	Cigarette and hookah (n: 41)	*p* value
FBG^1^ (mg/dl)	92.65 ± 38.71	96.78 ± 43.26	87.59 ± 33.86^ab^	93.60 ± 42.43^c^	94.85 ± 47.02	0.013
TG^2^ (mg/dl)	117 (83–170)	128 (90.5–177.5)	122 (86–180)	121 (88–171)	122 (96–186)	0.125
Cholesterol^1^ (mg/dl)	192.41 ± 39.36	191.10 ± 38.76	184.18 ± 39.4^a^	190.53 ± 37.6^a^	189.27 ± 37.25	0.045
LDL^1^ (mg/dl)	116.77 ± 35.53	117.18 ± 35.45	112.42 ± 34.93	117.48 ± 33.63	109.03 ± 35.39	0.9
HDL^1^ (mg/dl)	43.63 ± 10	41.96 ± 10.18	38.77 ± 9.22^ab^	42.53 ± 9.04^ab^	41.11 ± 9.65	< 0.001
Uric acid^1^ (mg/dl)	4.62 ± 1.4	4.91 ± 1.45	5 ± 1.34^ab^	4.36 ± 1.32^c^	5.37 ± 1.6^c^	< 0.001
Serum urea^1^ (mg/dl)	12.93 ± 4.28	13.54 ± 4.2	13.48 ± 4.22	12.52 ± 4.05	13.8 ± 5.57	0.63
Cr^1^ (mg/dl)	0.85 ± 0.25	0.90 ± 0.24	0.93 ± 0.25	0.84 ± 0.27	0.93 ± 0.21	0.65
GFR^3^ ml/min						
< 60	119 (2.7%)	28 (4.4%)	33 (5.7%)	10 (1.5%)	1 (3.3%)	< 0.001
> 60	4364 (97.3%)	605 (95.6%)	546 (94.3%)	646 (98.5%)	29 (96.7%)	
hsCRP^2^ (mg/l)	1.62 (0.98–3.51)	1.55 (1–3.35)	1.71 (1.06–3.89)	1.63 (1.04–3.46)	2.02 (1.1–4.65)	< 0.001

^a^No-smoker vs ex-smoker, cigarette, hookah

^b^Ex-smoker vs cigarette, hookah

^c^Cigarette vs hookah

^1^Mean ± SD

^2^Median (min–max)

^3^Number (%)

Table 2 shows the association between hematological parameters and smoking status. After adjustment for age and sex, WBC (p < 0.001), RBC (p < 0.001), Hb (p < 0.001), HCT (p < 0.001), MCV (p < 0.001), MCH (p < 0.001), PLT (p = 0.004) and RDW (p < 0.001) were significantly different but MCHC (p = 0.065) was not different with respect to smoking status.

**Table 2:** Association between smoking status and CBC parameters (adjusted for age and sex)

Groups	No smoker (n: 6742)	Ex-smoker (n: 976)	Cigarette smoking (n: 864)	Hookah smoking (n: 1067)	Cigarette and hookah (n: 41)	*p* value
WBC (×10^3^/µl)	5.95 ± 1.46	6.16 ± 1.58^a^	6.97 ± 1.98^ab^	6.05 ± 1.51^ac^	6.58 ± 1.86^ad^	< 0.001
RBC (×10^6^/µl)	4.82 ± 0.47	4.95 ± 0.57	5.04 ± 0.49^ab^	4.8 ± 0.47^abc^	4.93 ± 0.84^d^	< 0.001
Hb (mg/dl)	13.57 ± 1.53	14.14 ± 1.46^a^	14.76 ± 3.01^a^	13.42 ± 1.61^b^	14.16 ± 2.22	< 0.001
HCT (%)	40.81 ± 5.32	42.37 ± 4.56	43.79 ± 3.67^a^	40.52 ± 3.99^a^	42.41 ± 7.14	0.001
MCV (fl)	84.61 ± 6.09	85.49 ± 5.78^a^	86.96 ± 5.64^ab^	84.47 ± 5.95^bc^	86.19 ± 3.78	< 0.001
MCH (pg)	28.24 ± 2.73	28.6 ± 2.23	29.16 ± 2.24^ab^	27.98 ± 2.68^c^	28.67 ± 1.74	< 0.001
MCHC (g/dl)	33.23 ± 1.65	33.3 ± 1.43	33.49 ± 1.25	33.07 ± 1.57	33.32 ± 1.11	0.065
PLT (×10^3^/µl)	231.43 ± 60.29	227.37 ± 53.12^a^	210.85 ± 51.48^b^	238.55 ± 68.78^c^	236.31 ± 67.15^c^	0.004
RDW (%)	41.42 ± 3.18	41.92 ± 3.11^a^	42.83 ± 3.14^ab^	41.9 ± 3.33^ac^	42 ± 2.56	< 0.001

Values expressed in mean ± SD

^α^No-smoker vs ex-smoker, cigarette, hookah

^b^Ex-smoker vs cigarette, hookah

^c^Cigarette vs hookah

After adjustment for age and sex, smoking status was significantly associated with the presence of CVD (p < 0.001), obesity (p < 0.001), metabolic syndrome (p < 0.001), diabetes (p < 0.001), and dyslipidemia. Table 3 shows these relationships.

**Table 3:** Association between smoking status and CVD, obesity, metabolic syndrome, diabetes and dyslipidemia (adjusted for age and sex)

Groups	No smoker (n: 6742)	Ex-smoker (n: 976)	Cigarette smoking (n: 864)	Hookah smoking (n: 1067)	Cigarette and hookah (n: 41)	*p* value
CVD n (%)						
Yes	819 (12.3%)	184 (19.0%)	115 (13.6%)	136 (12.9%)	4 (10.0%)	< 0.001
No	5847 (87.7%)	782 (81.0%)	733 (86.4%)	921 (87.1%)	36 (90.0%)	
Obesity n (%)						
Normal weight	1714 (25.5%)	285 (29.2%)	414 (48.0%)	216 (20.3%)	10 (25.0%)	< 0.001
Over weight	2904 (43.1%)	423 (43.4%)	331 (38.4%)	424 (39.9%)	23 (57.5%)	
Obese	2111 (31.4%)	267 (27.4%)	117 (13.6%)	423 (39.8%)	7 (17.5%)	
Metabolic syndrome n (%)						
Yes	2611 (38.8%)	398 (40.8%)	227 (26.2%)	497 (46.8%)	14 (34.1%)	< 0.001
No	4113 (61.2%)	577 (59.2%)	637 (73.7%)	566 (53.2%)	27 (65.9%)	
Diabetes n (%)						
Yes	925 (14.0%)	180 (18.9%)	82 (9.8%)	180 (17.1%)	6 (16.2%)	< 0.001
No	5693 (86.0%)	772 (81.1%)	755 (90.2%)	875 (82.9%)	31 (83.8%)	
Dyslipidemia n (%)						
Yes	4394 (65.5%)	691 (70.9%)	640 (75.1%)	709 (67.1%)	33 (80.5%)	< 0.001
No	2312 (34.5%)	278 (29.1%)	212 (24.9%)	348 (32.9%)	8 (19.5%)	

Results of multivariate analysis are shown in Table 4. Only PLT and RDW remained significant in the cigarette or hookah smokers in comparison with non-smokers. While HDL, MCV and WBC and remained significant only in the cigarette smokers in comparison with the non-smokers. Glucose and WBC were significantly different between ex-smokers and non-smokers. Moreover, CVD was significant higher for ex-smokers and current cigarette smokers in comparison with non-smokers. The presence of obesity and metabolic syndrome was positively associated with hookah smoking and negatively associated with cigarette smoking. Dyslipidemia was associated with hookah smoking only in this sample (Table 4).

**Table 4:** The relative risk of being an ex-smoker, or hookah and cigarette smoking associated with biochemical measurements, CBC parameters and different metabolic disorders; the reference group is non-smokers

Groups	Reference group and ex-smoker	Reference group and cigarette	Reference group and hookah	Reference group and cigarette and hookah
FBG	1.003 (1.001–1.005)***	0.999 (0.995–1.002)	0.999 (0.995–1.002)	1.005 (0.996–1.014)
Cholesterol	1 (0.998–1.003)	1.002 (0.999–1.005)	1.005 (0.989–1.010)	0.997 (0.985–1.009)
Uric acid	0.939 (0.998–1.015)	0.921 (0.823–1.007)	0.898 (0.823–0.979)***	1.114 (1.1–1.307)*
Hs-CRP	1.007 (0.998–1.016)	1.005 (0.994–1.016)	1.041 (0.991–1.067)	1.019 (0.991–1.048)
HDL	0.998 (0.986–1.009)	0.978 (0.965–0.991)***	0.923 (0.865–1.007)	1.001 (0.995–1.049)
GFR	1.013 (0.655–1.567)	0.78 (0.51–1.19)	1.21 (0.627–2.35)	1.064 (0.139–8.15)
WBC	1.145 (1.069–1.225)***	1.509 (1.406–1.629)***	1.34 (0.906–1.242)	1.140 (0.849–1.513)
RBC	1.079 (0.425–2.733)	0.642 (0.298–1.386)	0.865 (0.453–1.34)	2.854 (0.27–30.151)
HGB	0.993 (0.709–1.391)	1.235 (0.960–1.589)	1.15 (0.934–1.789)	0.873 (0.357–2.135)
HCT	1.005 (0.988–1.022)	1.005 (0.972–1.039)	1.015 (0.989–1.139)	1.010 (0.93–1.097)
MCV	1.051 (1–1.103)*	1.077 (1.009–1.148)*	1.047 (0.109–1.248)	1.380 (1.126–1.692)**
MCH	0.918 (0.765–1.101)	0.845 (0.714–1)	0.945 (0.814–1.32)	0.636 (0.395–1.024)
PLT	1 (0.999–1.002)	0.996 (0.994–0.998)**	0.996 (0.785–1.098)*	1.002 (0.995–1.010)
RDW	1.008 (0.969–1.048)	1.089 (1.043–1.138)***	1.103 (1.003–1.148)*	0.923 (0.777–1.096)
CVD	1.605 (1.335–1.931)***	1.42 (1.13–1.79)**	1.022 (0.838–1.24)	0.991 (0.347–2.83)
Obesity^a^				
Obese	1.021 (0.847–1.23)	0.474 (0.377–0.597)***	1.31 (1.096–1.572)**	1.018 (0.38–2.72)
Over weight	0.937 (0.794–1.105)	0.57 (0.483–0.673)***	1.096 (0.918–1.307)	1.61 (0.76–3.41)
Metabolic syndrome	1.17 (1.013–1.35)*	0.82 (0.69–0.97)*	1.29 (1.12–1.48)***	1.134 (0.58–2.19)
Diabetes	0.79 (0.65–0.95)*	1.14 (1.12–1.88)**	0.75 (0.63–0.9)**	0.71 (0.56–1.42)
Dyslipidemia	0.93 (0.8–1.08)	0.87 (0.73–1.03)	0.82 (0.71–0.94)**	0.57 (0.26–1.25)

Adjusted odds ratios with age and sex, 95% confidence intervals (95% CI) obtained from multiple logistic regression tests. * p < 0.05, ** p < 0.01, *** p < 0.001

^a^Reference is normal BMI

## Discussion

The current study investigated the effect of hookah and cigarette smoking on the presence of CVD, obesity, MetS, diabetes mellitus, dyslipidemia and some biochemical and hematological parameters. To the best of our knowledge this is the largest study exploring the adverse effect of hookah smoking. In line with a recent study in Saudi Arabia FBG was not significantly associated with hookah smoking [10] while cigarette smokers had significantly lower levels of glucose in compare with non-smokers. The few studies on the effect of hookah smoking on lipid profile are inconsistent. Our results show significantly lower levels of cholesterol and HDL in hookah smokers in comparison to non-smokers, while TG and LDL was not different between these two groups. A similar study with 152 subjects including 75 cigarette smoker, 77 hookah smoker and 16 healthy controls did not show any significant relationship between hookah smoking and levels of plasma lipids [6]. While, Koubaa et al. [14] with 68 subjects reported lower levels of HDL in hookah smokers in comparison with non-smokers. They also showed a significant increase in serum TG in hookah smokers, which we did not find. We did not find any differences in GFR and uric acid in hookah smokers in comparison with non-smokers while cigarette smoking significantly associated with impaired GFR. Furthermore, serum Cr and urea were not significantly different in hookah nor cigarette smokers compared to non-smokers.

Hookah smokers had significantly lower levels of RBC, HCT and higher levels of WBC and PLT in comparison with non-smokers. In other words, we found that the effect of hookah and cigarettes on RBC and HCT was in the opposite direction. Moreover, HB and RBC levels were similar in combined cigarette and hookah smokers as in non-smokers. This is not consistent with some previous studies. Miri-Moghaddam et al. [9] found that chronic exposure of rats to water pipe smoke was associated with a significantly increase in RBC and HCT whilst not affecting Hb, WBC and PLT. In a case–control study from Sudan, hookah smokers had significantly higher levels of Hb, HCT, RBC and WBC while there was no association between hookah smoking and PLT [8].

Surprisingly, we found the effects of hookah smoking were greater with respect to some CVD risk factors compared with cigarette smoking. The association of hookah smoking on the prevalence of obesity, metabolic syndrome and diabetes mellitus were in the opposite direction from cigarette smoking. Obesity, metabolic syndrome, diabetes and dyslipidemia were positively associated with hookah smoking while negatively associated with cigarette smoking. Interestingly the prevalence of obesity, metabolic syndrome and diabetes subjects who smoked both cigarette and hookah was less than hookah smokers and more than cigarette smokers. Moreover, the prevalence of dyslipidemia which was positively associated with hookah and cigarette smoking individually, was higher in subjects who smoked both hookah and cigarettes. Our findings are in line with the results of the population based study of 2032 individuals. In this study it was found that hookah smoking was positively related to metabolic syndrome and almost all components of metabolic syndrome [15]. Another study on the adverse effects of hookah, indicated the positive relation between hookah and diabetes [16].

## Conclusion

In contrast with the public belief that hookah eliminates the toxicity of tobacco in compare with cigarette we found that the adverse effects of hookah smoking could be even greater than cigarette. Prevalence of obesity, metabolic syndrome, dyslipidemia and diabetes was significantly higher in hookah smokers in compare with non-smokers and even cigarette smokers.
